# Prevalence and genotype distribution of viral hepatitis B in Cambodia between 1990 and 2020: a systematic review and meta-analysis

**DOI:** 10.1186/s13690-022-00880-9

**Published:** 2022-04-13

**Authors:** Bunthen E, Pichetra Ou, Serge Ouoba, Md Razeen Ashraf Hussain, Ko Ko, Shintaro Nagashima, Aya Sugiyama, Tomoyuki Akita, Junko Tanaka

**Affiliations:** 1 Department of Epidemiology, Infectious Disease Control and Prevention, Graduate School of Biomedical and Health Sciences, Hiroshima University, 1-2-3, Kasumi, Minami-ku, 734-8551 Japan; 2 Payment Certification Agency (PCA), Ministry of Health, Phnom Penh, Cambodia; 3 Fertility Clinic of Cambodia, Phnom Penh, Cambodia; 4 Unité de Recherche Clinique de Nanoro (URCN), Institut de Recherche en Sciences de La Santé (IRSS), Nanoro, Bobo-Dioulasso, Burkina Faso

**Keywords:** Hepatitis B, Genotype, Prevalence, Epidemiology, Cambodia

## Abstract

**Background:**

Hepatitis B virus (HBV) infection is one of the major public health problems globally as well as in Cambodia. Continuous information on HBV infection burden is required to implement effective disease control strategies. This study aimed to determine the prevalence and genotype distribution of HBV infection in Cambodia through a systematic review with meta-analysis.

**Methods:**

Four databases (PubMed, Web of Science, Scopus, and Google Scholar) were used to search published studies reporting either HBV prevalence or genotype distribution in Cambodia until August 21, 2020. Reviews, modeling studies, and studies conducted among Cambodian permanently living abroad were excluded. The Freeman–Tukey double arcsine transformation was implemented to achieve approximate normality. The DerSimonian and Laird method was used to compute pooled estimates based on the transformed values and their variance. Possible publication bias was assessed by the Egger test and the funnel plot.

**Results:**

A total of 22 studies were included, covering 22,323 people. Ten studies reported HBV prevalence in the general population. The HBV infection prevalence was 4.73% (95%CI: 2.75–7.17%) in the general population and 19.87% (95%CI: 10.95–30.63%) in high-risk/co-infected groups. By sub-group analysis, the prevalence was 6.81% (95% CI: 4.43–9.66) in adults older than 15 years old, 2.37% (95% CI:0.04–7.05) in children 6–15 years old, and 2.47% (95% CI: 0.96–4.59) in children less than five years old. The prevalence of HBV infection decreased over time. Predominant HBV genotypes were genotypes C and B with 82.96% and 16.79%, respectively.

**Conclusions:**

The decrease in HBV infection prevalence in Cambodia demonstrates the effects of national hepatitis B immunization, improved clinical hygiene, and the use of disposable devices. However, the estimated HBV prevalence among the general population indicates an intermediate endemicity level of HBV infection. Therefore, population screening and linkage to care, high vaccination coverage, health promotion, and HBV surveillance are essential to meet the WHO 2030 goal.

**Supplementary Information:**

The online version contains supplementary material available at 10.1186/s13690-022-00880-9.

## Background

Hepatitis B virus (HBV) infection is a major public health problem globally. The virus that causes hepatitis B can be transmitted from mother to child, direct contact with infected blood or other body fluids, sexual intercourse with an infected person, or sharing sharp instruments. In 2015, the number of people living with chronic HBV infection was estimated at 257 million, and 887 000 died mainly as the result of hepatocellular carcinoma (HCC) [1]. Therefore, at the 69^th^ world health assembly in 2016, 194 member states committed to eliminate viral hepatitis by 2030 [2].

Cambodia is located in the World Health Organization (WHO) Western Pacific Region, which is reported to have the highest prevalence of HBV infection (6.2% of the adult population) among all WHO regions [1]. The WHO Western Pacific region bears over 45% of the global HBV burden [3]. However, the prevalence of HBV infection varies considerably among state members, ranging from 1.0% to 18.8% [3]. HBV infection is one of the public health burdens in Cambodia. Children infected with HBV before five years old are more likely to develop chronic infections [1]. Therefore, administration of the hepatitis B vaccine (Hep B) within 24 h of birth, followed by three doses of pentavalent vaccine (at 6, 10, and 14 weeks of life), have been introduced into Cambodia’s national immunization program since 2005 [4]. Prior to the implementation of Hep B vaccine, the prevalence of HBV infection was reported at 2.02% (95% CI 0.27–3.77%) among children born between 1999 to 2005 in Siem Reap province [5]. The latest large-scale nationwide study on the prevalence of HBV infection in 2017 found a prevalence of 0.56% (95% CI 0.32–0.98%) among children aged 5–7 years, and 4.39% (95% CI 3.53–5.45%) in their mothers [6].

Cambodia has made a tremendous effort to combat HBV by achieving the 2017 target of lowering the HBV prevalence among children to less than 1 percent [4]. Eliminating mother-to-child transmission is another milestone on which the government of Cambodia is focusing. Moreover, some tasks must be done to move towards the global target of hepatitis B elimination by 2030, including boosting the awareness program, prevention, and treatment strategy. To implement effective strategies, more information on HBV infection is needed. This systematic review and meta-analysis aimed to estimate the prevalence of HBV infection and its genotype distribution in Cambodia.

## Methods

### Literature and search strategy

Four literature databases, PubMed, Web of Science, Scopus, and Google Scholar were used, and the last search was conducted on August 21, 2020. The keywords were: hepatitis B, hepatitis B surface antigen, HBV, HBsAg, epidemiology, prevalence, seroprevalence, DNA, genotype, and Cambodia. The search was modified to match the particular structure of each database (Supplementary Table 1).

### Study selection

We included all the published studies reporting HBV infection prevalence or genotype distribution among people living in Cambodia or Cambodian temporally living abroad. However, we excluded reviews, modeling studies, studies conducted among Cambodian permanently living abroad or duplicated articles that studied the same population during the same period.

All studies extracted from literature databases were reviewed independently by two reviewers (BE & PO). First, the duplicated records were removed using the Endnote reference manager. Then, the title and abstract were screened to eliminate irrelevant studies, and the full texts of potentially eligible studies were assessed for inclusion. Furthermore, the snowballing approach was used to identify additional studies. The final inclusion decision was based on consensus or by a third reviewer (AS) if no consensus was reached.

The following information was extracted independently by two reviewers (BE & PO): year of the study, year of publication, first author, study design, study population, study setting, diagnostic method, sample size, number of positive cases, and genotype distribution.

### Quality assessment

The quality of each included study was assessed separately by two reviewers (BE & PO) following the Guideline of Recommendation Assessment, Development and Evaluation (GRADE) guideline [7]. The quality of evidence was classified into four categories (high, moderate, low, and very low quality).

### Data management and analysis

EndNote X9 was employed to manage citations. Extracted data were recorded in Microsoft Excel and analyzed with STATA® 16, using the ‘metaprop’ package. Given that the included studies were conducted at different periods, on different population groups, and in different geographic locations, a random-effect model was employed because of the expected heterogeneity across studies [8, 9]. The Freeman–Tukey double arcsine transformation was implemented to achieve approximate normality. Then, the DerSimonian and Laird method was used to compute the pooled estimates based on the transformed values and their variance. The Wald method was then used to calculate the confidence intervals for the pooled estimates [9]. The Cochran’s Q test was used to determine heterogeneity, and the I^2^ index was used to quantify it. When the *p*-value of the Cochran’s Q test was less than 0.05, heterogeneity was considered significant. I^2^ of ≥ 50% was considered as substantial heterogeneity.

Among the general population, subgroup analysis was performed to minimize the heterogeneity and for group comparison. A *p*-value less than 0.05 was considered statistically significant.

The Egger test and funnel plot were used to assess possible publication bias.

### Study guideline

This systematic review was conducted following the 2020 Preferred Reporting Items for Systematic Reviews and Meta-Analysis (PRISMA) guidelines [10]. (Supplementary Table 2 and 3).

## Results

### General overview

**Fig. 1:**
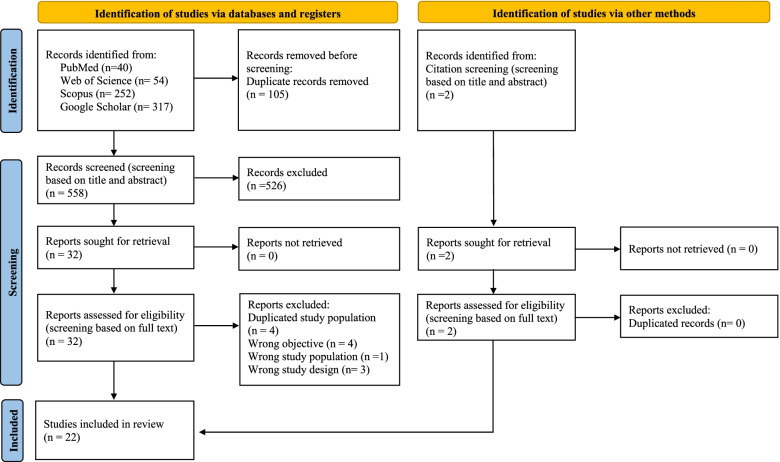
Flow chart of selecting published studies through database searching. At first, 663 studies were identified via databases, and 2 studies were identified via citation screening. After abstract screening and full-text assessment, 22 studies were included in the quantitative synthesis

A total of 663 studies were identified through database search. After removing duplicates, followed by titles and abstracts screening, 32 studies were eligible for full text and bibliography assessment. Out of them, 20 studies met the inclusion criteria, and two additional studies were included from manual citation screening. Thus, the quantitative analysis included 22 studies that covered 22,323 people. (Fig. 1). The summary of all included studies is presented in Table 1.

**Table 1:** Summary of the studies on prevalence or genotype of HBV in Cambodia, 1990–2020

No	Study Period	Publication Year	First Author	Study Design	Study Population	Study Area	Grade	Diagnostic Method	Number of participants with HBV infection	Total number of participants	Genotype
1	1990–1991	1993	Thuring E. G	Cross-sectional	General population	Rural	Medium	Abbott, North Chicago, USA	41	505	-
2	1996–1998	2002	Chhour Y. M	Cross-sectional	General population	Urban	Low	AUZYME Monoclonal	1	44	-
3	1997	2003	Sarmati, L	Cross-sectional	General population	Rural	Low	Serodia Hbs; Fujirebio, Inc	15	164	-
4	2006	2009	Soeung S. C	Cross-sectional	General population	Nationwide	High	Abbott Determine test strip; Abbott Laboratories, Abbott Park, IL	55	1558	-
5	2007	2009	Ol H. S	Cross-sectional	General population	Rural	Medium	Monolisa® BioRad	92	1200	-
6	-	2010	Sa-Nguanmoo P	Cross-sectional	General population	Rural	Medium	Murex Biotech Limited, Dartford, Kent, England	121	1119	A = 1, B = 13, C = 86
7	2011	2013	Mao B	Cross-sectional	General population	Rural/Urban	High	Alere Determine™	34	2402	-
8	2010–2012	2015	Yamada H	Cross-sectional	General population	Rural	Medium	Reversed Passive hemagglutination assay (R-PHA)	22	483	-
9	2011–2015	2018	Fujimoto M	Pro-cohort	General population	Rural	High	Mycell II HBsAg; Tokyo Japan; Architect HBsAg QT; Abbott, Tokyo, Japan; Lumipulse; Fujirebio, Tokyo, Japan	5	248	-
10	2017	2017	Ork V	Cross-sectional	General population	Nationwide	High	Alere Determine™	103	4546	-
11	1997–1998	2000	Ohshige K	Cross-sectional	High risk/co-infection	Rural	Medium	Serodia, Fujirebio, Tokyo, Japan	19	202	-
12	2002	2004	Buchy P	Cross-sectional	High risk/co-infection	Urban	Low	Monolisa HBsAg Plus®	37	90	-
13	2003–2012	2014	van Griensven, J	Retro-cohort	High risk/co-infection	Urban	High	Roche Diagnostics, Mannheim Germany Abbott laboratories, Illinois, US	341	3098	-
14	2006–2011	2015	Narin P	Cross-sectional	High risk/co-infection	Urban	Low	N/A	113	209	-
15	2003–2015	2016	Chassagne, F	Cross-sectional	High risk/co-infection	Urban	Medium	N/A	221	511	-
16	2014–2016	2017	De Weggheleire A	Cross-sectional	High risk/co-infection	Rural	Medium	Roche Diagnostics	311	3045	-
17	2014–2015	2018	Rouet, F	Cross-sectional	High risk/co-infection	Rural	Medium	Alere Medical Co, Chiba, Japan	27	209	-
18	2016–2017	2019	Nouhin, J	Pro-cohort	High risk/co-infection	Rural/Urban	High	N/A	51	2548	-
19	2001–2002	2006	Srey C. T	Case control	-	Urban	Medium	Abbott Laboratories, Chicago, IL, USA	-	22	B = 6, C = 16
20	2003	2008	Huy T. T	Cross-sectional	-	Urban	Medium	axsym HBsAg	-	12	B = 4, C = 8
21	2010–2014	2019	Chuon C	Pro-cohort	-	Rural	High	R-PHA, Mycell II HBsAg; Institute of Immunology, Tokyo Japan; Architect HBsAg QT; Abbott, Tokyo, Japan	-	26	B = 2, C = 24
22	2017	2020	Ko K	Cross-sectional	-	Nationwide	High	LumipulseII® HBsAg, Fujirebio, Japan	-	82	B = 16, C = 66

*N/A* Not applicable, *Pro-cohort* Prospective cohort study, *Retro-cohort* Retrospective cohort study

Seventeen studies reported only the prevalence of HBV infection [5, 6, 11–25], four studies reported only HBV genotype distribution [26–29], and one study reported both the prevalence and genotype distribution of HBV infection [30]. Among the studies that reported HBV prevalence, ten were conducted among the general population, and eight investigated the high-risk/co-infection population. (Table 1).

### Overall prevalence estimate

**Fig. 2:**
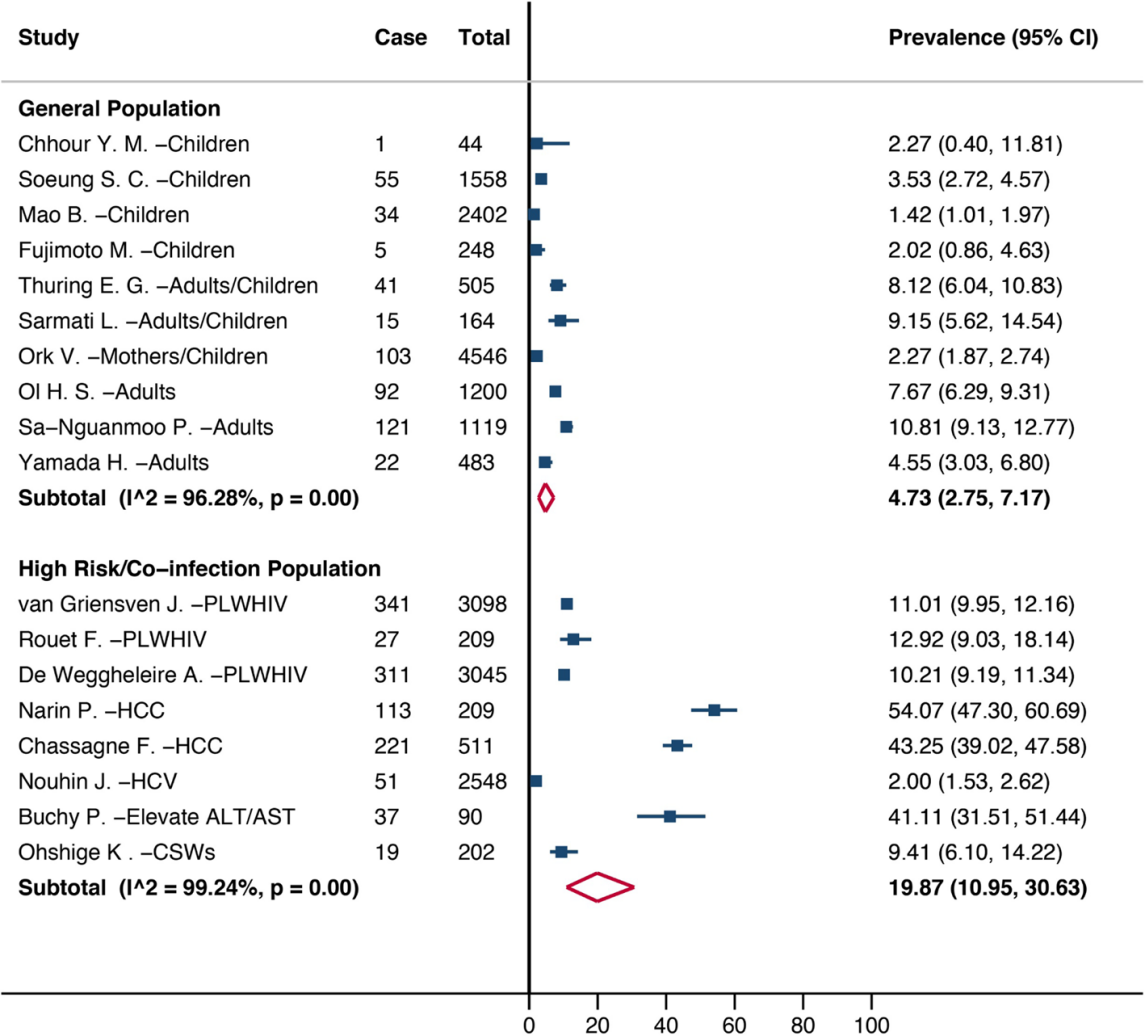
Estimated prevalence of HBV infection in Cambodia, 1990 and 2020. This figure shows the pooled prevalence of HBV infection in Cambodia in the general and high-risk/co-infection populations. The blue square presents the prevalence of HBV infection in each individual study with its 95% confidence interval. The red diamond represents the pooled prevalence of HBV infection in each population group. PLWHIV: People Living with HIV; HCC: Hepatocellular Carcinoma; HCV: Hepatitis C Virus; ALT/AST: Alanine Aminotransferase/Aspartate Aminotransferase

Of the 18 prevalence studies, 10 studies were conducted among the general population between 1990 to 2017 [5, 6, 13, 15, 19, 21–23, 25, 30]. In the general population, HBV infection pooled prevalence was 4.73% (95% CI: 2.75–7.17%), ranging from 1.42% (95% CI: 1.01–1.97%) to 10.81% (95% CI: 9.13–12.77%), with large heterogeneity across studies (I^2^ = 99.23%, Cochran’s Q test *p* < 0.001). Pooled HBV infection prevalence in high risk/co-infection population between 1998 to 2017 [11, 12, 14, 16–18, 20, 24] was 19.87% (95% CI 10.95–30.63%), ranged from 2% (95% CI: 1.53–2.62%) to 54.07% (95% CI: 47.30–60.69%), with large heterogeneity across studies (I^2^ = 99.23%, Cochran’s Q test *p* < 0.001). (Fig. 2).

### Prevalence of HBV infection among the general population based on participants’ age

Nine studies provided age information of the subjects, and subgroup analysis was conducted in three age categories: less than 5 years (4 studies), 6–15 years (4 studies), > 15 years (5 studies). The estimated HBV infection prevalence in adults older than 15 years was 6.81% (95% CI 4.43–9.66%) and was higher than the other age groups (*p* = 0.022). (Table 2).

**Table 2:** Sub-group analysis of HBV prevalence among general population in Cambodia, 1990 to 2020

Group	Sub-group	Prevalence (%) (95% CI)	Test for subgroup differences *p*-value	I^2^ Index (%)	Cochran’s Q test *p*-value	Number of Study
Age						
	Children (≤ 5 years-old)	2.47 (0.96–4.59)	0.022	92.92	< 0.001	4(6, 15, 22, 23)
	Children (6–15 years-old)	2.37 (0.04–7.05)		98.85	< 0.001	4(5, 6, 13, 23)
	Adults (> 15 years-old)	6.81 (4.43–9.66)		92.12	< 0.001	5(6, 19, 23, 25, 30)
Study Area						
	Rural	5.98 (3.50–9.06)	0.032	93.67	< 0.001	7(5, 15, 19, 21, 23, 25, 30)
	Urban	0.04 (0.00–0.39)		N/A	-	2(13, 15)
	Nationwide	2.56 (2.17–2.97)		N/A	-	2(6, 22)
Study Period						
	Before 2005	7.58 (5.31–10.19)	0.020	N/A	-	3(13, 21, 23)
	From 2005	3.32 (1.84–5.19)		94.82	< 0.001	6(5, 6, 15, 19, 22, 25)

*N/A* Not applicable

### Prevalence of HBV infection among the general population based on study area

Six of the ten studies on the general population were conducted exclusively in rural areas [5, 19, 21, 23, 25, 30]. Two studies were performed nationwide[6, 22], one study was conducted in urban areas [13], and one study was conducted in both urban and rural areas [15]. The estimated prevalence was 5.98% (95% CI: 3.50–9.06%), 0.04% (95% CI: 0.00–0.39%) and 2.56% (95% CI: 2.17–2.97%) among studies conducted in rural areas, urban areas and nationwide, respectively. (Table 2).

### Prevalence of HBV among the general population based on study period

The prevalence of HBV infection among the general population in Cambodia was also estimated based on the study period. Three studies were conducted before 2005 [13, 21, 23], and six were conducted after 2005 [5, 6, 15, 19, 22, 25]. One study published in 2010 did not specify the samples collection period [30]. The results showed that the prevalence of HBV infection decreased over time: 7.58% (95% CI: 5.31–10.19%) for the studies conducted before 2005 and 3.32% (95% CI: 1.84–5.19%) for the studies conducted after 2005 (*p* = 0.020). (Table 2).

### Genotype distribution

**Fig. 3:**
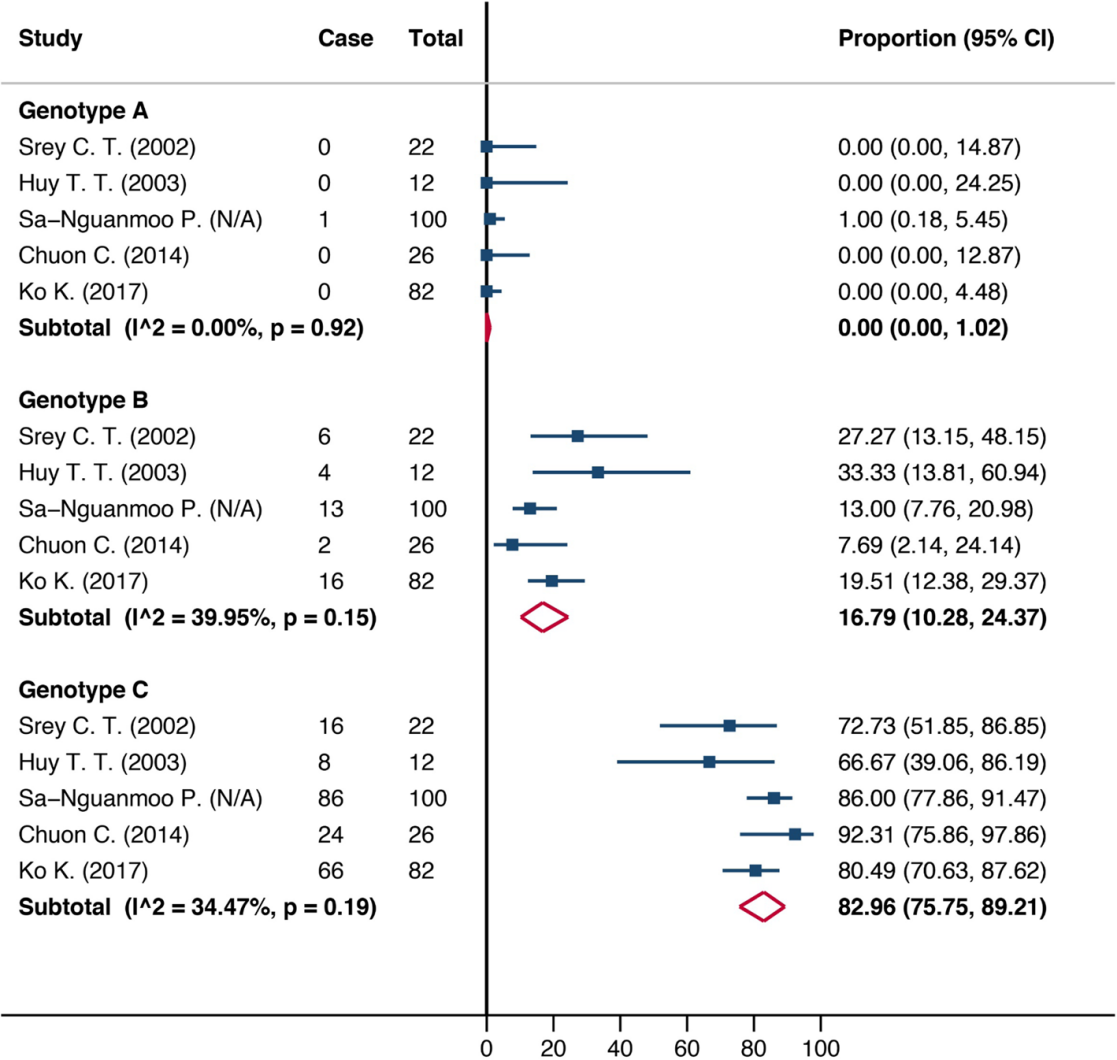
HBV genotype distribution in Cambodia, 1990 and 2020. This figure shows the distribution of HBV genotypes in Cambodia from the studies published between 1990 and 2020. The blue square presented the proportion of genotype in each individual study with the 95% confidence interval. The red diamond represents the pooled proportion of each genotype. N/A: not applicable

Five studies reported HBV genotype distribution in Cambodia [26–30]. They were published between 1990 and 2020 with a sample size range of 12 to 100. Of the eight well-known genotypes of HBV (A-H), genotypes A, B, and C were identified. The predominant HBV genotype was type C (82.96%). (Fig. 3).

### Publication bias

The Egger test showed no evidence of publication bias, with a *p*-value = 0.20. The funnel plot also illustrated a distribution of HBV prevalence studies among the general population in Cambodia close to symmetry. (Supplementary Fig. 3).

## Discussion

Endemicity of hepatitis B infection is classified into three levels: high (≥ 8% of HBsAg prevalence), intermediate (2–7% of HBsAg prevalence), and low (< 2% of HBsAg prevalence) [31]. Our estimated HBV prevalence of 4.73% (95% CI: 2.75–7.17%) in the Cambodian general population classifies the country among those with intermediate endemicity. This estimate is similar to the previously reported prevalence of 4.39% (95% CI: 3.53%–5.45%) among mothers of 5–7 years old children in the 2017 nationwide study [6]. Compared to neighboring countries at a similar period, our reported HBV prevalence is lower (5.1–6.42% in Thailand, 10.79–11.2% in Vietnam, and 8–8.74% in Laos) [32–35].

In countries with intermediate HBV endemicity, both horizontal and vertical transmission routes exist, but vertical transmission represents the main transmission mode [31]. Therefore, HBV screening among pregnant women to identify those at risk of transmitting it to their babies, and implementation of prevention measures in childhood can remarkably reduce the spread of the virus and the future occurrence of HCC.

Based on participants’ age, the prevalence of HBV infection among adults (> 15 years old) was about three folds significantly higher than that among children. We believe that the difference was due to a cohort effect. Indeed, all those older than 15 were born before 2005, the year of implementation of the nationwide Hep B vaccine at birth in Cambodia, and most of them had not received the birth dose. Therefore, they were at a higher risk of HBV infection than those born after 2005, especially if they were born to a chronic HBV mother. Correspondingly, by year of study, HBV infection prevalence was higher in studies conducted before 2005 (7.58%) than those conducted after 2005 (3.32%). Health care waste management (HCWM) issued by the Ministry of Health of the Kingdom of Cambodia in July 2008 might have contributed to decreasing HBV prevalence, alongside vaccination programs [36]. These guidelines define all types of health care waste and the requirements for their identification, labeling, and classification. Also, the technical requirements for the segregation, collection, storage, handling, and disposal of waste generated by healthcare facilities are described. The HCWN regulations have certainly contributed to protecting health care workers, waste handlers, and the community from exposure to injury or infection, including hepatitis B. Moreover, disposable devices for invasive procedures (syringes, small surgery kits, ear-piercing, needles) have become more available in the last decade. Therefore, unsafe medical practices such as reusing syringes/needles for multiple patients would spontaneously diminish. In a nutshell, the difference in HBV prevalence among the two cohorts can be explained by the direct and indirect impact of the nationwide Hep B vaccination program, the improvement of clinical hygiene, HCWM, and use of disposable devices in Cambodia.

Our review found a higher prevalence of HBV infection in rural areas than in urban areas. But the included studies conducted in urban areas were performed only on the children population, while the studies conducted in rural areas were performed on both children and adults. Therefore, the lower HBV prevalence in urban areas could result from an imbalance in the study population (Supplementary Fig. 1). However, it is noteworthy that the estimated prevalence of HBV infection among children (≤ 15 years old) was higher in rural areas than in urban areas (3.88% vs 0.04%) (Supplementary Fig. 2). Even though the number of studies in each area was small, this difference might be explained by the Hep B vaccination coverage. It has been reported that the coverage of at least three doses of Hep B vaccine varied by region and was higher in urban areas (91%) than in rural (82%) and remote (64%) areas [15]. Nonetheless, the possibility of different risk factors across the two areas cannot be ruled out. Further studies on socio-behavioral factors affecting HBV transmission in different areas are needed.

We noticed that the prevalence of HBV infection decreased over time. In 2017, Cambodia achieved the national and regional goal of reducing hepatitis B prevalence in children to less than 1% [4]. These are the results of improved clinical hygiene, increased use of disposable products, and expanded coverage of the nationwide Hep B vaccination program since 2005. Twelve years later, Vichit O., et al. reported that 78.4% of children aged 5–7 years received the birth dose of Hep B vaccine, and 92.3% of them received three doses of the pentavalent vaccine [6]. However, only 5.97% of their mothers had ever received the Hep B vaccine [37].

HBV genotypes B and C are predominant in the Asia Pacific region [38]. In our study, all the five studies describing HBV genotypes in Cambodia consistently reported the predominance of genotype C, which is a known factor for HBeAg positivity and risk of HCC [39].

There were some limitations in this study. The inconsistency of diagnostic methods and different test generations might have influenced the estimated prevalence. Also, with the limited access to grey literature in Cambodia, our review was performed among published studies indexed in the four databases searched, which may not be comprehensive. Therefore, the calculated prevalence might be higher/lower than the actual prevalence. However, the results of the Egger test and the funnel plot showed no publication bias. Furthermore, some of the included studies had a small sample size. As a result, the DerSimonian and Laird estimator of the between-study variance might have bias [40–42]. The inclusion of some low-grade studies might have affected the pooled prevalence. Moreover, the number of studies reporting HBV genotypes in Cambodia between 1990 and 2020 was small, with limited information on the geographical location. Therefore, HBV genotype distribution by geographical location could not be performed.

To the best of our understanding, this is the first systematic review and meta-analysis of HBV prevalence and its genotype distribution in Cambodia. We followed the strict rules of the PRISMA reporting guidelines. Thus, the results of the present study give an insight into the prevalence of HBV infection in Cambodia. Future studies need to address these limitations to obtain more accurate results.

## Conclusion

This systematic review and meta-analysis addressed the variation of HBV prevalence and its genotype distribution in Cambodia. We observed that HBV prevalence decreased over time as a result of the implementation of Hep B vaccine, the improvement of clinical hygiene, and the use of disposable devices. However, the estimated HBV prevalence among the general population indicates an intermediate endemicity level of HBV infection. Therefore, population screening and linkage to care, high vaccination coverage, health promotion, and HBV surveillance are essential to meet the WHO 2030 goal.

## Supplementary Information

**Additional file 1: Supplementary Figure 1.** This figure shown the results of sub-groupanalysis among general population based on study area. The study area wasdivided into three groups, rural areas, urban area and nationwide. The bluesquare presented the prevalent of HBV infection in each individual study with95% confident interval. The red diamond showed the HBV infection pooledprevalent of each population group. **Supplementary Figure 2.** This figure shown the results of sub-group analysis among children aged equal or less than 15 years old based on study area. The study area was divided into three groups, rural areas, urban area and nationwide. The blue square presented the prevalent of HBV infection in each individual study with 95% confident interval. The red diamond showed the HBV infection pooled prevalent of each population group. **Supplementary Figure 3.** This figure shown the Funnel plot of arcsine of the estimate HBV infection prevalence by its reverse variance. Each blue dot represents the prevalence of each study. SE, standard error.

## Data Availability

All data generated or analyzed during this study are included in this published article.

## Abbreviations

ALT/AST: Alanine Aminotransferase/Aspartate Aminotransferase
ES: Effect Size
HBV: Hepatitis B Virus
HCC: Hepatocellular Carcinoma
HCV: Hepatitis C Virus
HCWM: Health Care Waste Management
PLWHIV: People Living with HIV
WHO: World Health Organization
